# A Phase 1 study of BAL101553, a novel tumor checkpoint controller targeting microtubules, administered as 48-h infusion in adult patients with advanced solid tumors

**DOI:** 10.1007/s10637-019-00850-z

**Published:** 2019-08-30

**Authors:** Markus Joerger, Anastasios Stathis, Yannis Metaxas, Dagmar Hess, Mara Mantiero, Michael Mark, Matthias Volden, Thomas Kaindl, Marc Engelhardt, Patrice Larger, Heidi Lane, Peter Hafner, Nicole Levy, Silvia Stuedeli, Cristiana Sessa, Roger von Moos

**Affiliations:** 1 Cantonal Hospital St. Gallen, St. Gallen, Switzerland; 2 Oncology Institute of Southern Switzerland, Bellinzona, Switzerland; 3 Department of Medical Oncology, Cantonal Hospital Graubünden, Chur, Switzerland; 4 Basilea Pharmaceutica International Ltd, Grenzacherstrasse 487, PO Box, CH-4005 Basel, Switzerland; 5 Swiss Group for Clinical Cancer Research, Bern, Switzerland

**Keywords:** BAL101553, Microtubule-targeting agent, Chemotherapy, Ovarian cancer

## Abstract

*Purpose* BAL101553, the prodrug of the microtubule-destabilizer BAL27862, previously showed signs of antitumor activity when administered as a 2-h infusion, but its use was limited by vascular toxicity. We investigated an alternative dosing strategy aimed at improving the safety profile of BAL101553. *Methods* This multicenter, open-label, Phase 1 dose-escalation study used a 3 + 3 design to determine the maximum tolerated dose (MTD), dose-limiting toxicities (DLTs), pharmacokinetics, and antitumor activity of BAL101553 administered as a 48-h IV infusion on Days 1, 8, and 15 of a 28-day cycle. Patients received oral BAL101553 on Days 15–21 of cycle 2 to assess oral bioavailability. *Results* BAL101553 was well tolerated at doses up to ≤70 mg/m^2^. Three grade 3 DLTs occurred: hypotension (70 mg/m^2^), hyponatremia and neutropenia (both 90 mg/m^2^). The MTD for 48-h IV BAL101553 was 70 mg/m^2^. At this dose level, the AUC for BAL27862 was 8580 ng.h/mL and the C_max_ was 144 ng/mL. No apparent dose-related effects on blood pressure were observed with 48-h BAL101553 IV infusion. BAL27862 oral bioavailability was >80%. *Conclusions* Continuous 48-h IV BAL101553 infusion achieved higher exposure of the BAL27862 active metabolite than a 2-h infusion at the RP2D and did not cause vascular toxicity. Clinicaltrials.gov registration: NCT02895360.

**Electronic supplementary material:**

The online version of this article (10.1007/s10637-019-00850-z) contains supplementary material, which is available to authorized users.

## Introduction

Microtubule targeting agents (MTAs) are used therapeutically to induce either polymerization or depolymerization of microtubules and are categorized into two groups, known as stabilizers (including taxanes and epothilones) and destabilizers (including *Vinca* alkaloids, halichondrins and combretastatins) [1]. Microtubules are found in both interphase and dividing cells and play a key role in mitosis, intracellular trafficking, cell signaling, migration, secretion, angiogenesis, among other critical cell functions [1–3].

Stabilizing or destabilizing the microtubule polymer results in spindle assembly poisoning, mitotic blockage, and ultimately cell death through apoptosis [1]. However, despite many malignancies having a high initial sensitivity to MTAs, several mechanisms can result in drug resistance, including tumor overexpression of P-glycoprotein (Pgp), elevated levels of ß-tubulin subtype III, reduced levels of BRCA1 (the cancer susceptibility gene), elevated levels of the cell cycle inhibitory protein p21, and acquired mutations in ß-tubulin [4–9]. Consequently, there is a need to identify novel tubulin-inhibiting agents that overcome these resistance factors and improve treatment effectiveness.

BAL101553 (lisavanbulin; PubChem CID: 45259014) is a novel, synthetic molecule that has displayed promising antitumor activity in nonclinical studies. BAL101553 is a water-soluble lysine prodrug of BAL27862 (avanbulin; PubChem CID: 11176685), the active furazano-benzimidazole derivative [10, 11]. Nonclinical studies have shown that BAL27862 binds to microtubules at the colchicine site [11], leading to activation of the “spindle assembly checkpoint” with an associated block to mitotic progression and induction of apoptosis [12]. Detailed biochemical studies have shown that BAL27862 has a unique mechanism of action on microtubule dynamics, which is distinct from existing MTAs [11] and may explain its broad activity in a number of in vitro tumor models refractory to standard MTAs through both Pgp- and non-Pgp-related mechanisms [13, 14]. BAL101553 has also shown significant antitumor activity, after both IV and oral administration, across a panel of tumor xenograft models, including models refractory to standard MTAs as well as other standard of care therapies [15–23]. Moreover, synergistic activity has been observed in xenograft models [17–19, 23] with pathologically confirmed cures in some cases [21, 22]. Importantly, BAL101553 treatment not only has a profound effect on tumor cell proliferation and viability but can also target the tumor vasculature, indicative of a dual mechanism of action on refractory tumor cells and vascular cells [23, 24].

A recent study looked at the dosing, safety and tolerability of IV BAL101553 in patients with advanced solid tumors (study CDI-CS-001) [25]. This was a two-part (dose escalation followed by dose expansion), open-label, Phase 1/2a study. Patients received BAL101553 given by 2-h IV infusion on Days 1, 8, and 15 of a 28-day cycle. At doses ≥45 mg/m^2^, BAL101553 was associated with either fully or partially reversible, dose-limiting neurological and myocardial side effects, including gait disturbance and myocardial injury (grade 3 troponin elevation and electrocardiogram changes including T-wave inversions). However, the recommended Phase 2 dose level of 30 mg/m^2^ was well-tolerated and showed signals of antitumor activity, including one patient with an ampullary carcinoma who had a partial response lasting for over 2 years [25].

The dose-limiting toxicities (DLTs) in CDI-CS-001 (2-h IV infusion) were determined by the vascular-disrupting effect of BAL101553 and were observed in direct relation to C_max_. As nonclinical data suggest that the antiproliferative effects of BAL101553 are related to the AUC of BAL27862 [10], study CDI-CS-003 was designed to investigate alternative dosing strategies aimed at achieving a higher dose intensity with less vascular toxicity. The adopted approach was an extended IV infusion time of 48 h, as pharmacokinetic (PK) modelling suggested administration of BAL101553 over this extended time period would result in C_max_ levels around 25% of those observed when the drug is given as a 2-h infusion. In addition, oral administration of BAL101553 was investigated during this study to provide information on the oral bioavailability of the drug.

## Methods

### Study design

The work reported here is from the completed Phase 1 part of an ongoing open-label, multi-center Phase 1/2a study of single agent BAL101553 administered as a 48-h IV infusion in adult patients with advanced solid tumors. The study was conducted in accordance with the Declaration of Helsinki and Good Clinical Practice. Institutional Review Boards/Ethics Committees at the three study sites and relevant authorities in Switzerland approved the study, and all patients provided written informed consent prior to study participation. The study is registered (clinicaltrial.gov: NCT02895360).

### Dose escalation

The Phase 1 dose-finding study aimed to determine the maximum tolerated dose (MTD) in patients with solid tumors. Dose escalation was conducted using a 3 + 3 titration design, with patients enrolled in sequential (escalating) dose levels comprising three to six patients and using a body surface area-adjusted dosing approach (see Online Resource 1). For each dose level, new patients were recruited and evaluated for safety, PK, pharmacodynamic (PD) effects, and for antitumor activity. The starting dose level of IV BAL101553 was 30 mg/m^2^ (based on the clinical experience and outcomes from study CDI-CS-001 in patients with advanced solid tumors who were administered BAL101553 as a 2-h IV infusion [25]). Dose increments of approximately 50% were planned from 30 mg/m^2^ onwards until the occurrence of a DLT in any patient, after which all subsequent dose levels were to be incremented by 33%. DLTs were generally defined as grade ≥4 hematological adverse drug reactions or grade ≥3 non-hematological adverse drug reactions occurring during cycle 1 (see Online Resource 2).

Patients were enrolled in sequential dose cohorts comprising three patients. If one out of three patients experienced a BAL101553 treatment-related DLT during cycle 1, the dose cohort was expanded to include up to three additional patients. Decisions on dose escalations were based on clinical review of all relevant available data from contemporaneous and previous dose cohorts. Dose escalation continued until the maximum administered dose (MAD) was achieved, defined as the dose level at which a DLT was observed during treatment cycle 1 in ≥33% of evaluable patients. The MTD was defined as the highest dose level below the MAD with an acceptable tolerability profile in at least six patients.

### Patients

Key inclusion criteria were age ≥18 years with histologically- or cytologically-confirmed advanced or recurrent solid tumor who failed standard therapy or for whom no effective standard therapy is available, measurable disease according to RECIST v1.1, an Eastern Cooperative Oncology Group [ECOG] performance status ≤1, life expectancy ≥12 weeks, and adequate organ and marrow function. Key exclusion criteria included peripheral neuropathy ≥CTCAE grade 2; systolic blood pressure ≥140 mmHg or diastolic blood pressure ≥90 mmHg; chemotherapy, radiotherapy, immunotherapy, or investigational agents within 4 weeks (2 weeks for single fraction of palliative radiotherapy, 6 weeks for nitrosoureas or mitomycin C), or anti-androgen therapy for prostate cancer (except for chemical castration with luteinizing hormone-releasing hormone analogues) within 6 weeks, prior to starting study drug. Patients taking more than two antihypertensive medications were also ineligible for study participation, as were those with significant cardiac disease or abnormalities or with a history of cerebral hemorrhage, cerebral aneurysm, or ischemic stroke; a history of transient ischemic attack within 24 months prior to screening.

### Study treatment

BAL101553 was administered as a 48-h IV infusion through an implantable venous access system (“PORT”) using an elastomeric pump (Baxter pump models 2C4711K or 2C1009KP/2C4009K) on Days 1, 8, and 15 of a 28-day cycle, except for Days 15–21 of cycle 2 when it was administered orally. Patients were hospitalized for up to 72 h on Days 1 of cycle 1 and 2, and up to 48 h on Day 21 of cycle 2, for serial PK sampling and safety monitoring. Patients could be discharged following the 30-h PK sample.

Following completion of cycle 1 MTD-relevant safety assessments, oral BAL101553 capsules were administered on study days 15–21 of cycle 2 in place of the Day 15 IV infusion to assess oral drug bioavailability. Oral BAL101553 was administered as hard capsules, each containing 1 mg or 5 mg of BAL101553. Capsules were taken once daily in the fasted state before breakfast. Patients in the cohorts receiving IV BAL101553 at doses of either 30 mg/m^2^ or 45 mg/m^2^ received 8 mg of oral BAL101553 daily (equivalent to an IV dose of 30 mg/m^2^; based on PK observed in study CDI-CS-002 in patients with advanced solid tumors administered BAL101553 as daily oral capsules [26]). Patients who received 70 mg/m^2^ or 90 mg/m^2^ of IV BAL101553 during dose escalation received equivalent daily oral doses of 12 mg or 15 mg BAL101553, respectively.

Treatment with IV BAL101553 could be continued beyond the second 28-day cycle until disease progression, occurrence of unacceptable toxicity or other criteria for discontinuation were met. For treatment to continue beyond cycle 2, efficacy assessments scheduled every 8 weeks had to be completed.

If a DLT occurred, BAL101553 was temporarily withheld until recovery to ≤CTCAE grade 1 or baseline and reintroduced at a lower dose-level if deemed adequate. Dose reductions or interruptions were also possible for non-DLT events. If all three doses of BAL101553 were administered within 28 days, cycle 1 was considered complete. Intra-patient dose escalation was permitted in patients who completed ≥2 cycles of BAL101553 without any grade ≥2 drug-related adverse events (AEs) if this was considered safe. Discontinuation criteria included disease progression and unacceptable toxicity or wish of the patient.

### Study objectives

The primary objectives of this Phase 1 study were to determine the MTD and to characterize DLTs of BAL101553 when administered as an IV infusion over 48 h on study Days 1, 8 and 15 of a 28-day treatment cycle in adults with advanced or recurrent solid tumors for whom standard therapy failed or no effective standard therapy is available.

Secondary objectives were to further evaluate the safety and tolerability of BAL101553 when administered as a 48-h continuous IV infusion, to assess the drug’s antitumor activity, and to assess the PK of BAL101553 and BAL27862 following a 48-h IV infusion and after daily oral administration on study Days 15–21 of cycle 2 to estimate the bioavailability of BAL27862 when BAL101553 was administered orally.

### Safety assessments

Safety assessments were conducted throughout the study and included AEs according to CTCAE v4.03, serious adverse events, laboratory parameters, electrocardiogram, vital signs, Eastern Cooperative Oncology Group [ECOG] performance status, and physical examinations. Radiological assessments were conducted at screening, echocardiography at screening and the end of study, and concomitant medications were evaluated throughout the study.

### PK assessments

Blood samples for PK assessments were taken on Day 1 (pre-dose, 0.5, 1, 2, 4, 8, 24, 30, 48, 52, 54, and 72 h post-infusion start), and Day 8 and Day 15 (pre-dose, 1, and 48 h post-infusion start) of cycle 1; and on Day 1 (pre-dose, 0.5, 1, 2, 4, 8, 24, 30, 48, and 72 h post-infusion start), Day 15 (pre-dose), and Day 21 (pre-dose, 0.5, 1, 2, 4, 8, 24, 30, and 48 h after oral administration of BAL101553) of cycle 2. Additional blood samples were taken at the end of study, at the occurrence of a DLT, and on the first dosing day of the new dose level from patients undergoing dose escalation or reduction.

Liquid chromatography tandem-mass spectrometry with a lower limit of quantification of 1 ng/mL was used to quantify BAL101553 and BAL27862 levels in plasma. The relative bioavailability of active drug BAL27862 was determined as the ratio of the AUC following oral and IV administration. Preliminary PK calculations based on nominal times are reported here. Any measurement obtained from blood samples known or suspected to have been taken from the PORT used for study drug administration were excluded from the PK analyses as these samples could be contaminated with administered drug.

### Efficacy assessments

Efficacy was determined according to RECIST v1.1 criteria at baseline and within 7 days of completion of every second cycle to determine activity of study treatment. Patients with objective response or stable disease were permitted to continue treatment with BAL101553 until either disease progression or unacceptable toxicity. Radiology assessments were repeated at the End of Study Visit if they had not been performed within 28 days prior.

### Statistical analysis

All patients who received at least one partial or complete dose of BAL101553 based on the intent-to-treat principle formed the full analysis population (FAP). Safety was evaluated in all patients from the FAP who had at least one post-baseline safety assessment (safety population). The MTD population consisted of all patients who received all three doses of BAL101553 during cycle 1 (or ≥1 dose if the patient experienced a DLT) and were followed for ≥28 days after the first dose for safety.

Background, demographic, safety, PK and PD data were analyzed using descriptive statistics or contingency tables. Safety assessments were primarily based on the frequency of AEs and laboratory abnormalities among the safety population. Efficacy was evaluated in the FAP and the objective response rate and disease control rate were determined. The disease control rate was the proportion of patients with controlled disease (complete or partial response or stable disease) after two and four treatment cycles, and at the end of treatment.

## Results

### Patient demographics and disposition

Twenty patients (7 male, 13 female) were enrolled at three study sites across Switzerland between August 2016 and December 2017. The median age was 60.5 years and the patients had a median of three prior chemotherapies. There were four dose cohorts in this study, starting at 30 mg/m^2^ (*n* = 4), increasing to 45 mg/m^2^ (*n* = 3), 70 mg/m^2^ (*n* = 9) and 90 mg/m^2^ (*n* = 4). Baseline demographic and disease data are presented in Table 1.

**Table 1:** Baseline demographic and disease history data

	BAL101553 30 mg/m^2^	BAL101553 45 mg/m^2^	BAL101553 70 mg/m^2^	BAL101553 90 mg/m^2^	BAL101553 Total
Number of patients in FAP	4	3	9	4	20
Age, years, median (range):	53.0 (52–59)	61.0 (61–61)	61.0 (44–74)	61.5 (56–73)	60.5 (44–74)
Sex, n (%)					
Male	2 (50.0)	1 (33.3)	3 (33.3)	1 (25.0)	7 (35.0)
Female	2 (50.0)	2 (66.7)	6 (66.7)	3 (75.0)	13 (65.0)
Race, n (%)					
White	4 (100)	3 (100)	9 (100)	4 (100)	20 (100)
Primary active tumor, n (%)					
Bile duct	2 (50.0)	0	1 (11.1)	0	3 (15.0)
Breast	0	0	1 (11.1)	1 (25.0)	2 (10.0)
Breast upper inner quadrant	0	0	1 (11.1)	0	1 (5.0)
Esophagus	1 (25.0)	0	1 (11.1)	0	2 (10.0)
Lung	1 (25.0)	0	0	0	1 (5.0)
Ovary	0	1 (33.3)	3 (33.3)	1 (25.0)	5 (25.0)
Pancreas	0	0	1 (11.1)	1 (25.0)	2 (10.0)
Pleura	0	0	1 (11.1)	0	1 (5.0)
Uterus	0	1 (33.3)	0	0	1 (5.0)
Other	0	1 (33.3)	0	1 (25.0)	2 (10.0)
Tumor histology					
Adenocarcinoma	3 (75.0)	2 (66.7)	5 (55.6)	3 (75.0)	13 (65.0)
Other	1 (25.0)	1 (33.3)	4 (44.4)	1 (25.0)	7 (35.0)
Grade					
G1 (well differentiated)	0	0	0	2 (50.0)	1 (10.0)
G2 (moderately differentiated)	0	0	3 (33.3)	0	3 (15.0)
G3 (poorly differentiated)	2 (50.0)	2 (66.7)	1 (11.1)	0	5 (25.0)
Gx (grade not assessible)	2 (50.0)	1 (33.3)	5 (55.6)	2 (50.0)	10 (50.0)
Stage					
III	0	1 (33.3)	1 (11.1)	0	2 (10.0)
IV	4 (100)	2 (66.7)	7 (77.8)	4 (100)	17 (85.0)
Unknown	0	0	1 (11.1)	0	1 (5.0)
ECOG					
0	1 (25.0)	3 (100)	3 (33.3)	2 (50.0)	9 (45.0)
1	3 (75.0)	0	6 (66.7)	2 (50.0)	11 (55.0)
Prior tumor treatment, n (%)					
Chemotherapy or hormone therapy	4 (100)	3 (100)	9 (100)	4 (100)	20 (100)
Radiotherapy	2 (50.0)	0	3 (33.3)	1 (25.0)	6 (30.0)
Surgery	3 (75.0)	3 (100)	8 (88.9)	2 (50.0)	16 (80.0)

The median duration of exposure to BAL101553 was 43.5 days (mean 58 days). Of the 20 patients, 15 continued treatment in cycle 2 and nine patients received oral study medication. Treatment compliance was adequate: only three out of 126 infusions were incompletely administered, and four administrations were skipped during ongoing treatment due to AEs or patients wish. Two patients continued treatment at a reduced dose level after experiencing a DLT.

### Safety

IV BAL101553 was well tolerated at doses up to and including 70 mg/m^2^ and no DLTs were reported following administration of doses of 30 mg/m^2^ and 45 mg/m^2^. There were six MTD-evaluable patients among those treated at 70 mg/m^2^, one experienced a DLT of grade 3 hypotension in cycle 1 and one experienced a suspected unexpected serious adverse reaction of grade 3 peripheral neuropathy during cycle 2. Four patients were treated at 90 mg/m^2^, of which two experienced a DLT. The first experienced grade 3 hyponatremia and the second patient experienced grade 3 neutropenia, with grade 2 hallucinations and grade 2 ataxia, which led to dose reduction to 70 mg/m^2^. No relevant vascular toxicities were identified among any patient at any dose. All treatment-emergent related AEs are shown in Table 2.

**Table 2:** Treatment-emergent related adverse events by system organ class, preferred term and worst severity; safety population

System Organ Class / Preferred Term, n (%)	BAL101553 30 mg/m^2^ (*N* = 4)		BAL101553 45 mg/m^2^ (*N* = 3)		BAL101553 70 mg/m^2^ (*N* = 9)		BAL101553 90 mg/m^2^ (*N* = 4)		BAL101553 Total (*N* = 20)	
	Grade 1–2	Grade 3–4	Grade 1–2	Grade 3–4	Grade 1–2	Grade 3–4	Grade 1–2	Grade 3–4	Grade 1–2	Grade 3–4
All adverse events	2 (50.0)	0	2 (66.7)	0	6 (66.7)	2 (22.2)	1 (25.0)	2 (50.0)	11 (55.0)	4 (20.0)
Gastrointestinal disorders	1 (25.0)	0	0	0	4 (44.4)	0	3 (75.0)	0	8 (40.0)	0
Nausea	0	0	0	0	1 (11.1)	0	2 (50.0)	0	3 (15.0)	0
Abdominal pain	0	0	0	0	1 (11.1)	0	1 (25.0)	0	2 (10.0)	0
Diarrhoea	0	0	0	0	2 (22.2)	0	0	0	2 (10.0)	0
Abdominal distension	0	0	0	0	1 (11.1)	0	0	0	1 (5.0)	0
Vomiting	0	0	0	0	1 (11.1)	0	0	0	1 (5.0)	0
Stomatitis	1 (25.0)	0	0	0	0	0	0	0	1 (5.0)	0
General disorders and administration site conditions	0	0	0	0	5 (55.6)	0	3 (75.0)	0	8 (40.0)	0
Fatigue	0	0	0	0	4 (44.4)	0	3 (75.0)	0	7 (35.0)	0
Pyrexia	0	0	0	0	1 (11.1)	0	2 (50.0)	0	3 (15.0)	0
Metabolism and nutrition disorders	0	0	0	0	4 (44.4)	0	2 (50.0)	1 (25.0)	6 (30.1)	1 (5.0)
Decreased appetite	0	0	0	0	1 (11.1)	0	3 (75.0)	0	4 (20.0)	0
Hyponatraemia	0	0	0	0	2 (22.2)	0	0	1 (25.0) *	2 (10.0)	1 (5.0)
Dehydration	0	0	0	0	1 (11.1)	0	0	0	1 (5.0)	0
Hypokalaemia	0	0	0	0	1 (11.1)	0	0	0	1 (5.0)	0
Nervous system disorders	0	0	2 (66.7)	0	3 (33.3)	1 (11.1)	1 (25.0)	0	6 (30.0)	1 (5.0)
Peripheral sensory neuropathy	0	0	1 (33.3)	0	1 (11.1)	1 (11.1) *	0	0	2 (10.0)	1 (5.0)
Ataxia	0	0	0	0	0	0	1 (25.0)	0	1 (5.0)	0
Dysarthria	0	0	0	0	0	0	1 (25.0)	0	1 (5.0)	0
Headache	0	0	1 (33.3)	0	0	0	0	0	1 (5.0)	0
Neuralgia	0	0	0	0	1 (11.1)	0	0	0	1 (5.0)	0
Paraesthesia	0	0	0	0	1 (11.1)	0	0	0	1 (5.0)	0
Presyncope	0	0	0	0	1 (11.1)	0	0	0	1 (5.0)	0
Somnolence	0	0	0	0	1 (11.1)	0	0	0	1 (5.0)	0
Musculoskeletal and connective tissue disorders	1 (25.0)	0	0	0	0	0	2 (50.0)	0	3 (15.0)	0
Myalgia	1 (25.0)	0	0	0	0	0	1 (25.0)	0	2 (10.0)	0
Bone pain	0	0	0	0	0	0	1 (25.0)	0	1 (5.0)	0
Psychiatric disorders	0	0	0	0	0	0	2 (50.0)	0	2 (10.0)	0
Hallucination	0	0	0	0	0	0	2 (50.0)	0	2 (10.0)	0
Restlessness	0	0	0	0	0	0	1 (25.0)	0	1 (5.0)	0
Skin and subcutaneous tissue disorders	0	0	0	0	1 (11.1)	0	1 (25.0)	0	2 (10.0)	0
Alopecia	0	0	0	0	0	0	1 (25.0)	0	1 (5.0)	0
Hyperhidrosis	0	0	0	0	1 (11.1)	0	0	0	1 (5.0)	0
Infections and infestations	0	0	0	0	1 (11.1)	0	0	0	1 (5.0)	0
Lip infection	0	0	0	0	1 (11.1)	0	0	0	1 (5.0)	0
Injury, poisoning and procedural complications	0	0	0	0	1 (11.1)	0	0	0	1 (5.0)	0
Vascular access complication	0	0	0	0	1 (11.1)	0	0	0	1 (5.0)	0
Investigations	0	0	0	0	0	0	1 (25.0)	1 (25.0)	1 (5.0)	1 (5.0)
Blood creatinine increased	0	0	0	0	0	0	1 (25.0)	0	1 (5.0)	0
Neutrophil count decreased	0	0	0	0	0	0	0	1 (25.0) ^a^	0	1 (5.0)
Platelet count decreased	0	0	0	0	0	0	1 (25.0)	0	1 (5.0)	0
White blood cell count decreased	0	0	0	0	0	0	0	1 (25.0)	0	1 (5.0)
Vascular disorders	0	0	1 (33.3)	0	0	1 (11.1)	0	0	1 (5.0)	1 (5.0)
Hypertension	0	0	1 (33.3)	0	0	0	0	0	1 (5.0)	0
Hypotension	0	0	0	0	0	1 (11.1)	0	0	0	1 (5.0)

PT are coded according to MedDRA Version 17.0. Related means possibly related, probably related, or missing relationship (when relationship could not be determined from available source documents). A patient with multiple events within a PT is counted only once in the PT; the worst CTCAE grade is counted. ^a^ indicates DLTs

The MAD was 90 mg/m^2^. Following completion of safety and PK analysis along with other study assessments, 70 mg/m^2^ was identified as the MTD and established as the recommended Phase 2 dose (RP2D).

**Fig. 1:**
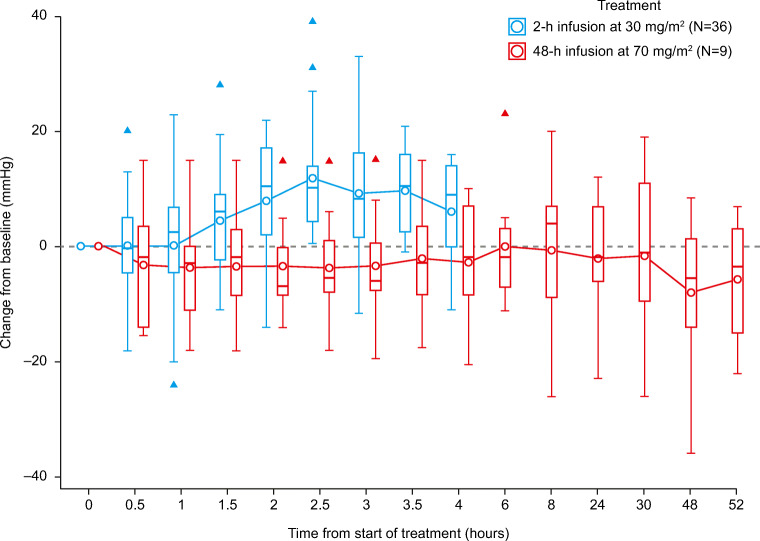
Average change from baseline in systolic blood pressure during cycle 1, Day 1. For comparison, the figure shows the rise in systolic blood pressure observed in study CDI-CS-001 where BAL101553 was administered as a 2-h infusion [25]. The figure shows data for patients who received BAL101553 at the MTD in study CDI-CS-003 (70 mg/m^**2**^; *n* = 9) and CDI-CS-001 (30 mg/m^**2**^; *n* = 36). The boxes represent the 25th to 75th percentile (interquartile range; IQR); solid lines are the medians; whiskers show the lowest / highest value within 1.5 × IQR (the box); and triangles represent outliers (values outside 1.5 × IQR). Arithmetic means (circles) are connected across time points

No effect on systolic blood pressure was observed after the first administration of BAL101553 when given as a 48-h IV infusion (Fig. 1).

### Pharmacokinetics

PK analysis demonstrated that the prodrug BAL101553 was rapidly converted to active BAL27862 in all patients and at all doses. Dose-related exposure with limited inter-individual variability was shown with both compounds and the AUC observed with BAL27862 was near dose-proportional.

**Fig. 2:**
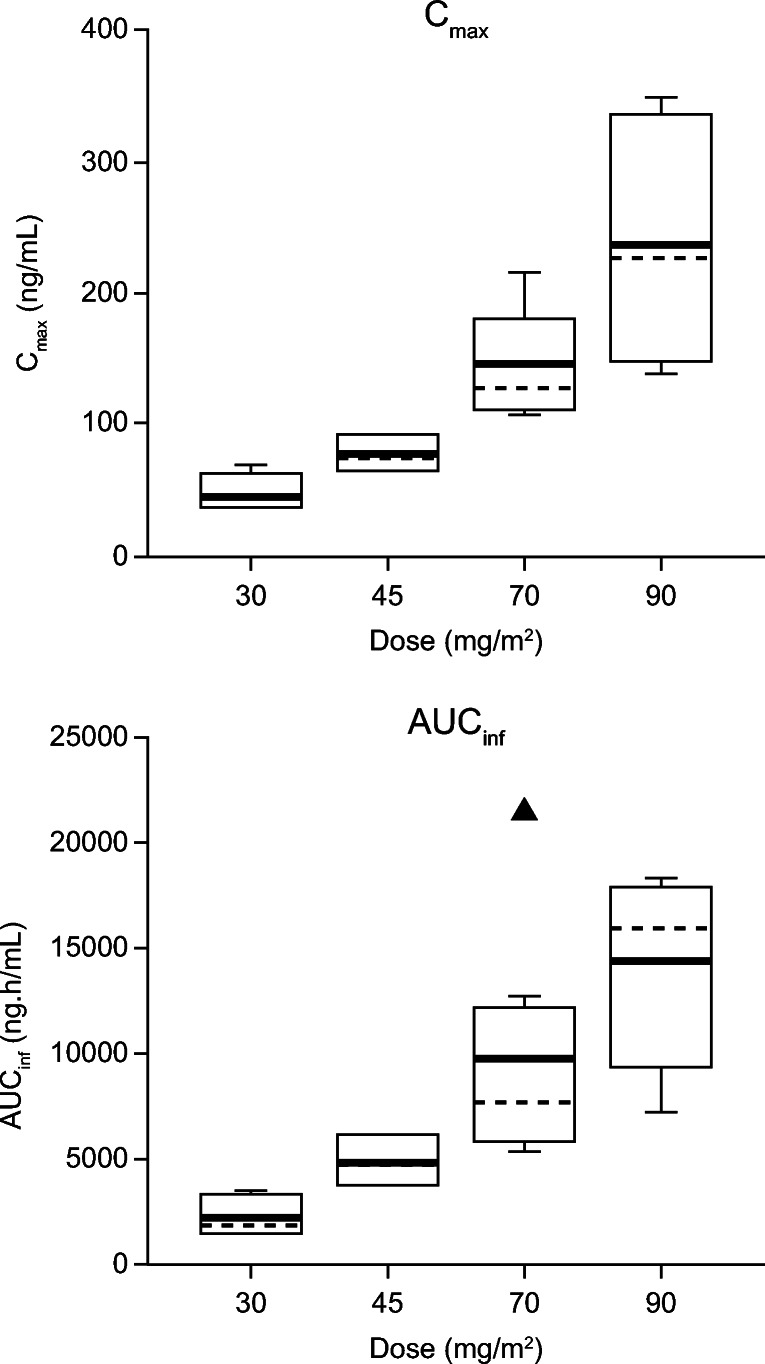
BAL27862 C_max_ and AUC_inf_ values for cycle 1 Day 1. For C_max_, *n* = 4, 3, 9, 4 at 30, 45, 70, 90 mg/m^2^; For AUC_inf_, *n* = 4, 2, 8, 4 at 30, 45, 70, 90 mg/m^2^. The boxes represent the 25th to 75th percentile (interquartile range; IQR); solid lines are the average (arithmetic mean); dashed lines are the median; whiskers show the lowest / highest value within 1.5 × IQR (the box); and individual points represents outliers (values outside 1.5 × IQR)

Steady state concentration was rapidly reached with BAL101553 whereas concentrations of BAL27862 increased steadily during the first 24 h. At the RP2D dose of 70 mg/m^2^, on cycle 1 Day 1 the BAL27862 C_max_ was 144 ng/mL and AUC was 8580 ng.h/mL (Fig. 2); the AUC/C_max_ ratio was 60.

**Fig. 3:**
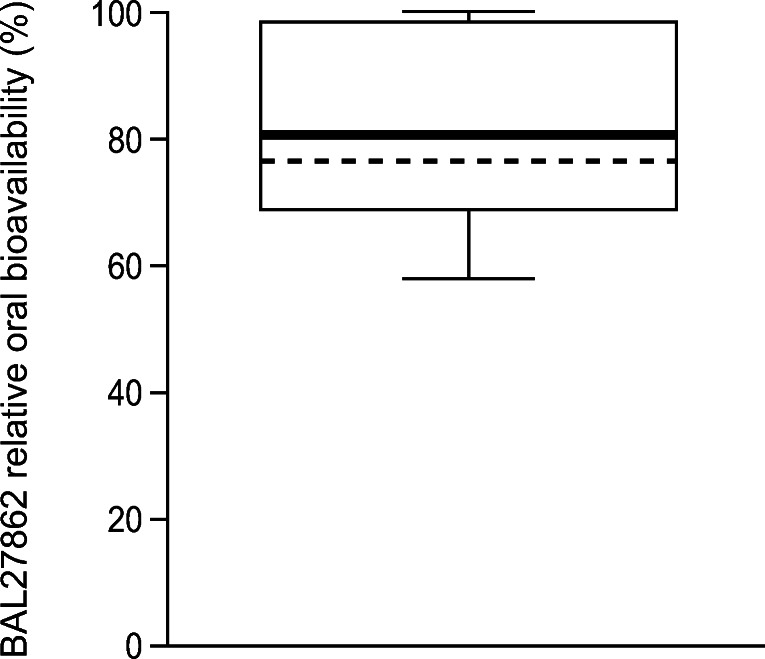
Estimated relative oral bioavailability* (*n* = 6). * Calculated as the ratio of dose-normalized AUC of BAL27862 after oral administration of BAL101553 to dose-normalized AUC of BAL27862 after IV administration of BAL101553

Daily oral BAL101553 at doses of 8, 12, or 15 mg/day was administered to patients for a 1-week period during cycle 2, from Day 15 through to Day 21. Following the last oral dose, the PK profile was collected; evaluable oral PK data were available from six patients. The relative oral bioavailability of BAL27862 was estimated to be >80% (Fig. 3), which is suggestive of an efficient delivery of active BAL27862 drug from oral BAL101553 prodrug.

### Efficacy

**Fig. 4:**
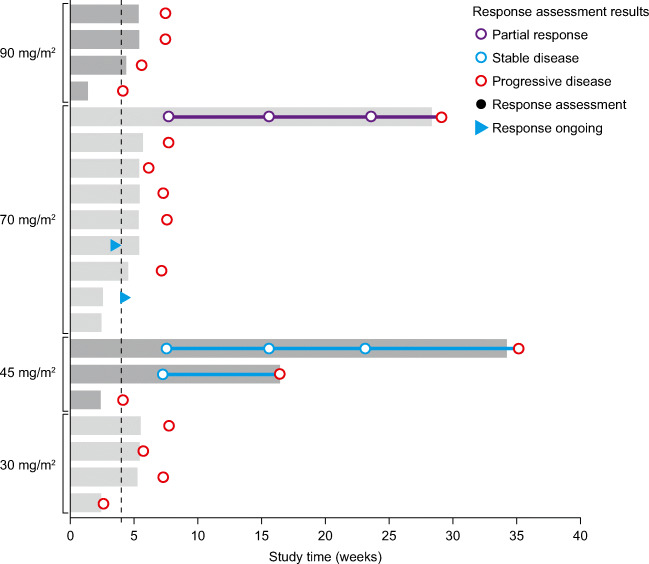
Swimmer plot showing duration on study and responses (*n* = 20). Dotted line at 4 weeks indicates the start of cycle 2. Two patients with responses (at least stable disease) ongoing (blue triangles) at the time of the last scan discontinued treatment due to adverse events or worsening of general condition

Of the 20 patients in study CDI-CS-003, 19 (95%) were evaluable for anti-tumor efficacy assessments (Fig. 4). One patient (treated at 70 mg/m^2^) was taken off study due to pneumonia in cycle 1 without a response assessment. In one patient with endometrial cancer treated at 45 mg/m^2^, lesions could not be tracked radiologically but the patient was assessed as clinically stable while being treated for nine cycles. One patient with ovarian cancer treated at 70 mg/m^2^ had a confirmed partial response following treatment for 150 days (21.4 weeks, 5.4 cycles). Therefore, based on radiological assessments at the end of cycle 2, the objective response rate was 1/20 (5%) and the disease control rate was 2/20 (10%).

## Discussion

We report the results of the Phase 1 open label study designed to determine the MTD of BAL101553 as a 48-h IV infusion in patients with solid tumors. After final analysis of all safety, tolerability, and PK data, 70 mg/m^2^ was identified as the MTD (RP2D) of BAL101553 administered as a 48-h infusion.

BAL101553 administered as a 48-h IV infusion was well tolerated up to and including the 70 mg/m^2^ RP2D. No DLTs were reported at the 30 mg/m^2^ and 45 mg/m^2^ doses. One DLT (grade 3 hypotension) was observed at the 70 mg/m^2^ dose. When the MAD of BAL101553 of 90 mg/m^2^ was administered, two of the four patients had DLTs: one patient experienced grade 3 hyponatremia and later discontinued due to disease progression, and the other experienced grade 2 visual hallucinations, grade 2 ataxia, and the only instance of grade 3 neutropenia; this led to a dose reduction.

The 48-h IV infusion of BAL101553 achieved a higher dose intensity and higher cumulative BAL27862 exposure, with lower vascular toxicity, compared with the 2-h infusion in study CDI-CS-001 [25]. The RP2D for the 48-h IV BAL101553 infusion was 70 mg/m^2^ versus 30 mg/m^2^ with the 2-h infusion. At these RP2D levels, the 48-h infusion achieved a higher mean AUC for the BAL27862 active drug (8580 vs 3620 ng.h/mL, respectively) while maintaining a lower mean BAL27862 C_max_ (144 vs 267 ng/mL, respectively). This resulted in a BAL27862 AUC/C_max_ ratio that was ~4-fold higher with the 48-h infusion compared with the 2-h infusion (60 vs 14, respectively).

There was no apparent vascular toxicity with the 48-h IV infusion of BAL101553. This is in contrast to study CDI-CS-001, where asymptomatic myocardial injury was observed in three patients at dose levels of ≥45 mg/m^2^ with a 2-h IV infusion [25]. There was also no apparent dose-related effect on blood pressure with the 48-h IV BAL101553 infusion, unlike in study CDI-CS-001 where arterial hypertension was significantly higher in patients treated with 60–80 mg/m^2^ compared with 15–30 mg/m^2^ BAL101553 administered as a 2-h IV infusion [25]. The 48-h IV infusion of BAL101553 at the RP2D did not increase systolic blood pressure during infusions, whereas transient elevations were observed with BAL101553 administered at the RP2D as a 2-h IV infusion [25]. The incidence and time course of blood pressure elevations in study CDI-CS-001 implied a C_max_-related vascular-disrupting effect of BAL101553. For this reason, we speculate that a prolonged infusion time with a lower C_max_ can diminish serious toxicity.

The incidences of other adverse events with the 48-h IV infusion were similar or less frequent than were observed with the 2-h infusion. Peripheral neuropathy occurred in three (15%) patients treated with BAL101553 administered as a 48-h IV infusion; however, peripheral neuropathy did not lead to any study withdrawals. Two of these events were grade 1/2 (one each at the 45 mg/m^2^ and 70 mg/m^2^ doses). In study CDI-CS-001, grade 2 peripheral sensory neuropathy with reduced proprioception/sensation was observed in two patients treated with 80 mg/m^2^ and was associated with the grade 2–3 gait disturbance DLTs in these patients [25]. At the RP2D for the 2-h infusion (30 mg/m^2^), two (5.6%) other patients experienced peripheral neuropathy, both grade 1/2 and reversible. Due to the axonal microtubule disrupting activity of MTA therapy, peripheral neuropathy may complicate MTA treatment [3, 9, 27].

The incidence of nausea, vomiting, and diarrhea, with 48-h IV infusions of BAL101553 at doses up to and including the RP2D of 70 mg/m^2^ was slightly lower than that observed with other MTAs [1].

Continuous drug infusions are a standard of care in oncology and well accepted by physicians and patients. This application is mostly used with 5-fluorouracil continuous intravenous infusion in patients with gastrointestinal tumors [28]. This study also investigated the pharmacokinetics of daily oral administration of BAL101553 in place of the IV infusion on study days 15–21 of cycle 2. Oral administration of BAL101553 may be more convenient and preferable to patients than continuous infusion. The administration of oral BAL101553 in the same patients showed good delivery of BAL27862 with a bioavailability of 80% relative to IV infusion, indicating that oral administration of BAL101553 may achieve the same benefits as the 48-h IV infusion. As the main aim of study CDI-CS-003 is to investigate 48-h IV dosing (including in the ongoing Phase 2a part of the study), oral administration of BAL101553 was only investigated during the third week of cycle 2. The intention was not to investigate the effects of switching from IV to oral dosing, rather to provide information on the oral bioavailability. Currently there are no approved oral MTAs. Daily continuous oral administration of BAL101553 is under investigation in patients with advanced solid tumors (including recurrent or progressive glioblastoma and high-grade glioma; CDI-CS-002, NCT02490800 [26]) and in combination with standard radiotherapy in patients with newly diagnosed glioblastoma (CDI-CS-004; NCT03250299).

The patient population of study CDI-CS-003 comprised patients for whom previous therapy had failed or no effective standard therapy was available. Following treatment with BAL101553 as a 48-h IV infusion, signals of efficacy were observed, consistent with those seen using the 2-h IV infusion in study CDI-CS-001 [25]. A patient with ovarian cancer had a confirmed partial response following treatment, and a second patient with endometrial cancer had stable disease lasting for nine cycles or 34 weeks. The ongoing Phase 2a part of study CDI-CS-003 aims to further characterize the safety and tolerability of BAL101553 administered as a 48-h IV infusion using the RP2D of 70 mg/m^2^ in patients with ovarian cancer or glioblastoma.

This study demonstrated that a 48-h continuous IV infusion of BAL101553 has a better tolerability profile than the previously explored 2-h infusion. The longer duration infusion achieved higher dose intensities with higher exposure of the active moiety BAL27862, without the C_max_-related vascular toxicities that were observed with the 2-h infusion. Other side effects were comparable or less intense than with the 2-h infusion of BAL101553. Clinical development of BAL101553 is ongoing.

## Electronic supplementary material

ESM 1 (PDF 523 kb)
