# Supplementary material for: A Phase 1 study of BAL101553, a novel tumor checkpoint controller targeting microtubules, administered as 48-h infusion in adult patients with advanced solid tumors
Source: Invest New Drugs. 2019 Aug 30;38(4):1067–76. doi: 10.1007/s10637-019-00850-z (PMC7340672; doi:10.1007/s10637-019-00850-z)
Supplement: Supplementary file 1 — (PDF 523 kb) [file 10637_2019_850_MOESM1_ESM.pdf]

Electronic supplementary material

Article title: A Phase 1 study of BAL101553, a novel tumor checkpoint controller, administered as 48-hour infusion in adult patients with advanced solid tumors

Journal: Investigational New Drugs

Authors: Markus Joerger, Anastasios Stathis, Yannis Metaxas, Dagmar Hess, Mara Mantiero, Michael Mark, Matthias Volden, Thomas Kaindl, Marc Engelhardt, Patrice Larger, Heidi Lane, Peter Hafner, Nicole Levy, Silvia Stuedeli, Cristiana Sessa, Roger von Moos.

Corresponding author: Thomas Kaindl, MD. Basilea Pharmaceutica Ltd, Grenzacherstrasse 487, PO Box, CH-4005 Basel, Switzerland; Tel: +41 (0)61 567 1505; E-Mail: Thomas.Kaindl@basilea.com

Online Resource 1: Dose-escalation criteria

Number of patients with DLT in the first 3 patients of a dose cohort [1]	Escalation decision
0 of 3 patients with DLT	Enroll 3 patients at the next dose level[2].
1 of 3 patients with DLT	• Enter 3 additional patients at this dose level.<br>– If only 1 of the 6 patients experiences a DLT, enter 3 patients at the next-higher dose level[2].<br>– If ≥ 2 of the [up to] 6 patients experience DLT, then dose escalation is stopped; this dose is declared the MAD. If only 3 patients were treated at the previous dose level, at least 3 additional patients must be enrolled at that dose to determine the MTD[2].
≥ 2 of 3 patients with DLT	Dose escalation must be stopped. This dose level will be declared the MAD. If only 3 patients were treated at the previous dose level, at least 3 additional patients must be enrolled at that dose to determine the MTD[2].

Maximum administered dose (MAD):
The MAD is the dose level with a rate of DLT in ≥ 33% of patients during treatment Cycle 1, i.e.:

- ≥ 2 of [up to] 3 patients with DLT in the first 3 patients of a dose level.
- ≥ 2 of [up to] 6 patients with DLT in a dose level that was expanded to 6 patients.

Maximum tolerated dose (MTD):
The MTD is the highest dose level below the MAD with an acceptable tolerability profile, i.e.:

- Not more than 1 of 6 patients with DLT at the highest dose level below the MAD.

At least 6 patients must be treated at the MTD level during the dose-escalation phase.
Intermediate dose levels may be assessed, e.g., if one dose is well tolerated without DLT and the subsequent dose level is defined as the MAD.

The number of patients refers to patients evaluable for DLT assessment. DLT=dose-limiting toxicity.
[1] Treatment related means causal relationship of the event to BAL101553 is considered to be at least 'possible'.
[2] Refer to Table 1 for descriptions of provisional dose levels.

Electronic supplementary material

Article title: A Phase 1 study of BAL101553, a novel tumor checkpoint controller, administered as 48-hour infusion in adult patients with advanced solid tumors

Journal: Investigational New Drugs

Authors: Markus Joerger, Anastasios Stathis, Yannis Metaxas, Dagmar Hess, Mara Mantiero, Michael Mark, Matthias Volden, Thomas Kaindl, Marc Engelhardt, Patrice Larger, Heidi Lane, Peter Hafner, Nicole Levy, Silvia Stuedeli, Cristiana Sessa, Roger von Moos.

Corresponding author: Thomas Kaindl, MD. Basilea Pharmaceutica Ltd, Grenzacherstrasse 487, PO Box, CH-4005 Basel, Switzerland; Tel: +41 (0)61 567 1505; E-Mail: Thomas.Kaindl@basilea.com

Online Resource 2: Overview of dose-limiting toxicities

Toxicity	CTCAE v4.03 criteria
Hematological	• Grade 4 Neutropenia (ANC < 0.5 × 10^9/L) lasting for ≥ 5 consecutive days.<br>• Febrile neutropenia (ANC < 1.0 × 10^9/L and single temperature of > 38.3 °C, or a sustained temperature of ≥ 38.0 °C for > 1 h).<br>• Grade 4 thrombocytopenia (platelet count < 25 × 10^9/L) or grade 3 thrombocytopenia (platelet count < 50 × 10^9/L) with bleeding.<br>• Any other ≥ grade 4 hematological AE.
Gastrointestinal	• ≥ Grade 3 nausea, vomiting or diarrhea despite appropriate pre-medication and/or management.
Hepatic	• Grade 3 AST or ALT elevations (> 5–20 × ULN) for > 7 days, or grade 4 (> 20 × ULN) for any duration.
Cardiac	• Grade 3 QTc interval prolongation (QTcF > 500 ms or > 60 ms change from baseline).<br>• Hypertension-related DLT:<br>– Grade 4 hypertension.<br>– Any recording of SBP > 220 mmHg or DBP > 110 mmHg.<br>– At least one observation of SBP ≥ 160 mmHg or DBP ≥ 100 mmHg that does not resolve to SBP < 160 mmHg and DBP < 100 mmHg within 24 hours, despite antihypertensive treatment.<br>Note: The need for administration of new antihypertensive medication or modification to more intensive antihypertensive medication will not be considered a DLT.
Other AEs	• Any study-treatment-related AE which leads to missing both the Day 8 and Day 15 doses in Cycle 1, or causes a delay in the start of Cycle 2 by > 14 days.<br>• Any other study-treatment-related AE which, in the view of the Investigator and/or Sponsor, represents a clinically significant hazard to the patient.<br>• Any other ≥ grade 3 non-hematological study-treatment-related AE.
Exceptions	• The following study-treatment-related AEs will not be considered DLT unless considered to present a clinically significant hazard to the patient:<br>– Grade 3 fatigue.<br>– Grade 3 or 4 elevations in alkaline phosphatase.<br>– Grade 3 or 4 hypophosphatemia.<br>– Grade 4 lymphopenia.
